# Multi-scale attention patching encoder network: a deployable model for continuous estimation of hand kinematics from surface electromyographic signals

**DOI:** 10.1186/s12984-024-01525-4

**Published:** 2024-12-30

**Authors:** Chuang Lin, Qiong Xiao, Penghui Zhao

**Affiliations:** The School of Information Science and Technology, Dalian Maritime University, Dalian, 116026 China

**Keywords:** sEMG, Hand kinematics, Continuous estimation, Multi-scale attention, Patching encoder, Transformer

## Abstract

**Background:**

Simultaneous and proportional control (SPC) based on surface electromyographic (sEMG) signals has emerged as a research hotspot in the field of human–machine interaction (HMI). However, the existing continuous motion estimation methods mostly have an average Pearson coefficient (CC) of less than 0.85, while high-precision methods suffer from the problem of long inference time (> 200 ms) and can only estimate SPC of less than 15 hand movements, which limits their applications in HMI. To overcome these problems, we propose a smooth Multi-scale Attention Patching Encoder Network (sMAPEN).

**Methods:**

The sMAPEN consists of three modules, the Multi-scale Attention Fusion (MAF) module, the Patching Encoder (PE) module, and a smoothing layer. The MAF module adaptively captures the local spatiotemporal features at multiple scales, the PE module acquires the global spatiotemporal features of sEMG, and the smoothing layer further improves prediction stability.

**Results:**

To evaluate the performance of the model, we conducted continuous estimation of 40 subjects performing over 40 different hand movements on the Ninapro DB2. The results show that the average Pearson correlation coefficient (CC), normalized root mean square error (NRMSE), coefficient of determination (R^2^), and smoothness (SMOOTH) of the sMAPEN model are 0.9082, 0.0646°, 0.8163, and − 0.0017, respectively, which significantly outperforms that of the state-of-the-art methods in all metrics (p < 0.01). Furthermore, we tested the deployment performance of sMAPEN on the portable device, with a delay of only 97.93 ms.

**Conclusions:**

Our model can predict up to 40 hand movements while achieving the highest predicting accuracy compared with other methods. Besides, the lightweight design strategy brings an improvement in inference speed, which enables the model to be deployed on wearable devices. All these promotions imply that sMAPEN holds great potential in HMI.

## Introduction

Myoelectric control provides intuitive control in human–machine interfaces (HMI) and has broad applications in various fields such as medical prosthetics and industrial control. Surface electromyographic (sEMG) signal is generated by muscle contractions beneath the skin and is directly related to human movement. It can be collected from shallow muscles through electrodes [1, 2]. Due to its abundant kinematic information and mature non-invasive acquisition techniques, sEMG has been widely applied in recognizing human movement intention, especially in hand movement recognition [3, 4].

However, sEMG signals are usually very weak, around µV (microvolts), making them susceptible to external environmental noise (such as electromagnetic interference) and internal physiological noise (such as electrocardiography and respiration) [3]. Due to physiological differences, exercise differences, psychological factors, etc., changes in sEMG signals occur among different subjects. Therefore, sEMG signals are usually preprocessed to overcome the noise. Besides, adaptive learning algorithms and deep learning models are used to overcome the influence of inter-subject variability and improve the quality and reliability of sEMG signals [2].

Traditional sEMG-based hand control methods use pattern recognition techniques. These techniques involve manual feature selection and machine learning algorithms. Although methods like Random Forests (RF) [5] and Support Vector Machines (SVM) [6] are successful, their performance may decrease as the demanded motion categories increase. To overcome these drawbacks, deep learning techniques are adopted [7–9]. However, methods based on discrete motion classification cannot capture continuous changes in human motion, which limits their applications. To address this challenge, researchers are focusing on continuous estimation of human movements, which is also known as simultaneous and proportional control (SPC). Surface EMG signals can estimate human movements more smoothly and naturally by establishing the continuous relationship between the sEMG signal and joint angle, angular velocity, joint torque, etc. [2].

Currently, continuous motion estimation methods based on sEMG signals can be mainly divided into model-based and model-free approaches [10]. Model-based methods involve constructing complex dynamic or musculoskeletal models to describe the relationship between sEMG and kinematic information. These methods have strong explanatory ability and accuracy, but face challenges such as complex model construction, difficult parameter measurements, and limited number of degree-of-freedoms that can be estimated [11, 12]. Model-free methods utilize artificial intelligence techniques to establish the relationship between sEMG and target values. Compared with model-based approaches, model-free methods have higher flexibility, making them more easily adopted in applications [13]. Deep learning methods have emerged as a powerful tool in the continuous estimation of myoelectric control in recent years. Most deep learning methods employing recursive structures, such as long short-term memory (LSTM) networks, capture temporal information from sEMG signals to fulfill the estimation [1, 14–17]. To capture the spatial and temporal features concurrently from sEMG signals, researchers combined LSTM with CNN [18–20]. Although the above models are state-of-the-art (SOTA) methods for sequence modeling, their Markov property constrains them to parallel train, which extends training time. The emergence of the temporal convolutional network (TCN) has alleviated the problem, but the prediction accuracy still needs to be improved [21]. Recently, Lin et al. [22] proposed a smoothed BERT model (sBERT) that utilizes a multi-head attention mechanism to achieve parallel training, which enhances prediction accuracy and training speed. However, having too many parameters in the model limits its deployment on portable devices.

Designing a model that has high accuracy, low latency, and strong deployment performance on wearable devices is the consistent target of sEMG-based motor intention estimation. In this paper, we proposed a smooth Multi-scale Attention Patching Encoder Network (sMAPEN) which allows parallel processing in the training stage. It comprises three modules: the Multi-scale Attention Fusion (MAF) module, the Patching Encoder (PE), and a smoothing layer. Inspired by [23, 24], we choose the combined feature as input, which is concatenated from three single features. The MAF can capture the local information from sEMG signals, while PEN acquires the global features. The smoothing layer is utilized to lessen fluctuations in the output. To validate this approach, we assessed our method with SOTA methods on the Ninapro dataset [5], which includes 40 subjects and 40 hand movements. The experimental results showed that our model exceeded SOTA methods in both accuracy and inference speed. The contribution of the paper can be summarized as follows:

- A MAF module is proposed to adaptively capture different scales of spatiotemporal features, and the extracted features are fused to improve the accuracy further. The details in Section II, Part A.
- The PE module is proposed to capture the global information while reducing the time complexity. The details are in Section II, Part B and C.
- A smoothing module is proposed to reduce fluctuations. The details are in Section II, Part D.
- The proposed model can predict up to 40 hand movements and achieves the highest predicting accuracy compared with other SOTA methods. The lightweight design strategy enables the model to be deployed on wearable devices, which means great potential in real applications.

## The proposed method

The Multi-Scale Attention Fusion (MAF) part uses three convolution operations with kernel sizes of 3, 5, and 7 to connect the ECA channel attention module. Then it adds up the results of the three operations to enhance the channel features of the input feature map while preserving the size.

The Patch Encoder (PE) section first divides the input data into multiple patches and adds positional information. Then, the Transformer Encoder extracts advanced features from the information to obtain the predicted joint angles.

The smoothing layer utilizes a small amount of historical data to process larger predicted values, to fit the actual human motion data.

### A. Features

To reduce latency and ensure an accurate representation of effective information, we employed a sliding window of 100-ms duration, with 0.5-ms stride length to extract features from each sEMG channel. We extracted the following features from these intervals. From the lightweight feature set, we selected three sEMG features [23]: Zero Order Moment, Shake Expectation, and Unbiased Standard Deviation. The formulas used for the calculation of these features are presented below:

#### 1) Zero Order Moment (ZOM)

Extracting the zero-order moment from time domain signals allows for the representation of muscle contraction strength through the square root of the zero-order moment. Adjusting the magnitude of its value using the logarithmic function brings it closer to the inverse characteristic. The expression for the square root characteristic of the electromyographic zero-order moment is denoted as ${m}_{0}$.

1 $$\begin{array}{*{20}c} {m_{0} = Log\left( {\sqrt {\sum\limits_{{i - 0}}^{{N - 1}} {x\left[ i \right]^{2} } } } \right)} \\ \end{array}$$

where $N$ represents the length of the sliding window, and $x\left[i\right]$, $i=1, 2,..., N$ represents the sEMG signal with a time length of $N$.

#### 2) Shake Expectation (SE)

In the field of sEMG processing, the expectation (Shaking Expectation, SE) of the EMG amplitude change speed can be expressed by the mean value of the absolute value of the second derivative. Calculated as follows:

2 $$\begin{array}{*{20}c} {SE = \frac{1}{N}\sum\limits_{{i = 0}}^{{N - 1}} {\left| {\Delta ^{2} x\left[ i \right]} \right|} } \\ \end{array}$$

where ${\Delta }^{2}$ represents the second derivative.

#### 3) Unbiased Standard Deviation (USTD)

Here, the unbiased standard deviation based on Bessel correction is used to reflect the degree of dispersion of the sEMG data. It is calculated as follows:

3 $$\begin{array}{*{20}c} {USTD = \sqrt {\frac{1}{N - 1}\sum\nolimits_{I = 0}^{N - 1} {\left| {x\left[ i \right] - \overline{x}} \right|^{2} } } } \\ \end{array}$$

where $\widehat{x}$ represents the expected value of the sEMG sample.

### B. Multi-scale Attention Fusion (MAF)

**Fig. 1:**
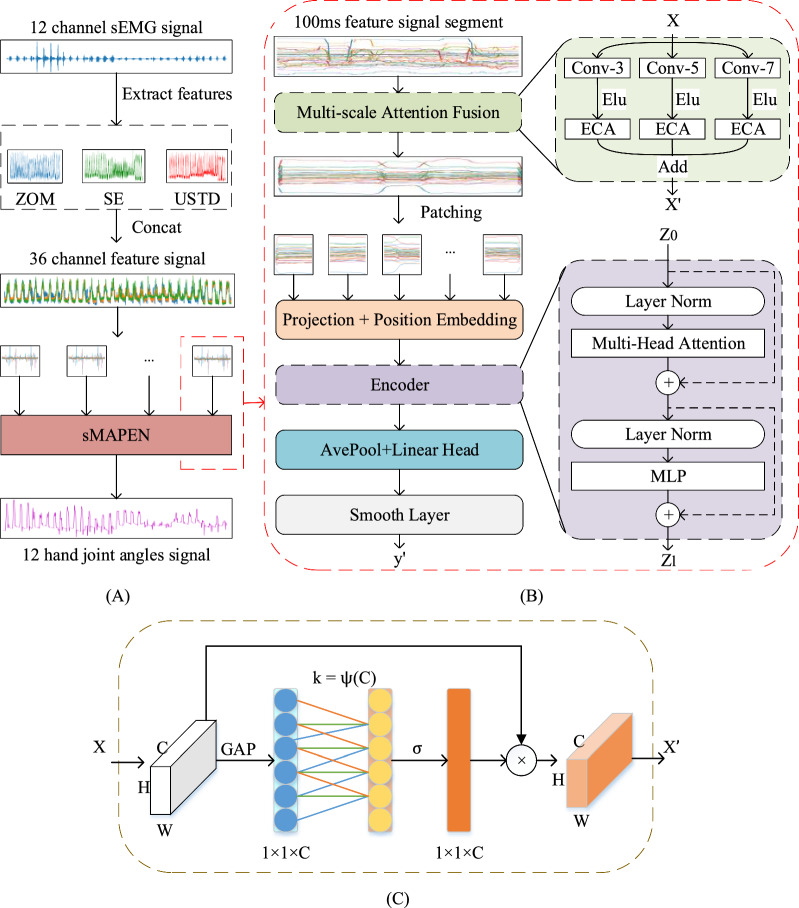
The model structure of the continuous hand movement estimation method based on sMAPEN for sEMG signal. **A** shows the overall flowchart, first extracting features from the raw sEMG, and then dividing the features into 100 ms (200 sampling points) slices for prediction by the model. Where ZOM, SE, and USTD denote three sEMG features: Zero Order Moment, Shake Expectation, and Unbiased Standard Deviation, respectively. “Concat” refers to the operation of splicing two matrices according to corresponding dimensions [24]. **B** is the specific structure of the sMAPEN model. Where Conv-3 represents convolutional computation using a convolutional kernel size of 3, Elu refers to the activation function, ECA represents the Efficient Channel Attention Module, and Add stands for computing sums [26]. **C** shows the structural details of the ECA module, where GAP represents global average pooling, k represents dynamic convolution kernel size, σ represents the activation function, and represents channel multiplication [25]

Essentially, the MAF module is the combination of multi-scale convolution and Efficient Channel Attention (ECA) modules [26] (Fig. 1). The kernel size of convolution is 3, 5, and 7 in this study. Multi-scale convolution is used to obtain the local temporal features. ECA employs a global average pooling technique to extract global features. A one-dimensional convolution operation is attached to ECA to learn inter-channel interactions. The kernel size of the one-dimensional convolution is crucial as it determines the interaction between channels. The kernel size* k* can be calculated by the following formula:

4 $$\begin{array}{*{20}c} {k = \psi \left( C \right) = \left| {\frac{1}{2}log_{2} \left( C \right) + \frac{1}{2}} \right|_{odd} } \\ \end{array}$$

where ${\mid t\mid }_{odd}$ represents the odd number closest to $t$. *C* denotes the channel dimension (i.e., number of filters).

In this study, we employ the ECA method to extract sEMG features between channels. This allows us to extract temporal scale information and inter-channel spatial information simultaneously. Finally, we combine the information from each feature branch to obtain a multi-scale feature representation $X^{\prime}\, \in \,{\mathbb{R}}^{W \times 1 \times C}$, which represents the extracted features:

5 $$\begin{array}{*{20}c} {X^{\prime} = F\left( {n_{1} } \right) + F\left( {n_{2} } \right) + F\left( {n_{3} } \right)} \\ \end{array}$$

6 $$\begin{array}{*{20}c} {F\left( n \right) = E\left( {\sigma \left( {f^{n \times 1;C} X} \right)} \right) } \\ \end{array}$$

where ${f}^{n\times 1; C}$ denotes *C* convolutional operations with $n\times 1$ kernels, $\sigma$ denotes the ELU activation function, and *E* denotes the ECA channel attention module. ${n}_{1}$*, *${n}_{2}$*,* and ${n}_{3}$ denote different convolutional kernel sizes, which are set to 3, 5, 7 in this paper.

### C. Patching Encoder (PE)

Patching Encoder (PE) consists of two processes: Patching Layer and Transformer Encoder. The patching layer divides the input data into multiple patches first, and after linear transformation, adds positional information as input information for the encoder. The Transformer Encoder consists of multi-head attention modules and multilayer perceptron modules, which can effectively extract advanced features of information and capture their complex relationships. And then ultimately obtaining predicted joint angles.

#### 1) Patching Layer

Before feeding the multi-scale feature fused signal $X^{\prime}$ into the encoder, we divide it into several non-overlapping patches, denoted as $X_{p} = \left\{ {x_{p}^{1} ,x_{p}^{2} \ldots x_{p}^{m} } \right\}$. The length of each patch is $C$, so the number of patches is $m=W/C$. Unlike traditional Transformer-based models, we do not treat each time point as a token, we treat each patch as a token. We linearly project each token to dimension d of the model through an embedding matrix $E$ and then fed it into a normal Transformer encoder directly. $E$ is randomly initialized while initializing the model. During the training process, each element is randomly selected and continuously optimized through a backpropagation algorithm and gradient descent to transform patches into more meaningful feature representations.

Since sEMG signal is a special type of time series, its sampling points at each time step do not have clear semantic meanings like words in a sentence. Therefore, extracting local semantic information is crucial for analyzing the relationships between them. Compared to many previous methods that only use token-level input point by point, we adopted the approach of aggregating the time steps into sEMG subsequence-level patches. This not only enhances locality but also captures comprehensive semantic information that is not available at the point level.

Additionally, dividing sEMG signals into fragment sequences brings another benefit: it can reduce the computational complexity so thus improving the efficiency of the model. The original attention mechanism has a time and space complexity of O(*N*^*2*^). However, by using patching, the number of input tokens can be reduced from input sequence length L to approximately $L/S$. This reduces the memory usage and computational complexity of the attention map by a factor of $S$, resulting in improved computational efficiency. Furthermore, to prevent overfitting, parameters in our prediction head will also be reduced by a factor of $S$.

What should be noted is that sEMG signals have a specific order, if the order is changed, the meaning of the input signal will also change accordingly. However, the transformer’s architecture does not model positional information, so the order of the input sequence needs to be encoded explicitly. In this study, we employ a learnable 1D position embedding matrix $E^{pos} \in {\mathbb{R}}^{n \times d}$ to capture position information. The learnable 1D position embedding matrix has the same dimension as patch embedding, and each row corresponds to the position information of a patch in the image. At the beginning of training, ${E}^{pos}$ will be randomly initialized, and then continuously optimized through gradient descent during the training process. Patch sequence have been generated with location information can be expressed as:

7 $$\begin{array}{*{20}c} {Z_{0} = \left[ {x_{p}^{1} E;x_{p}^{2} E; \ldots x_{p}^{m} E} \right] + E^{pos} } \\ \end{array}$$

where $x_{p}^{1} ,\,x_{p}^{2} \ldots x_{p}^{m}$ represents the patch partitioned from the input data, $E$ and ${E}^{pos}$ respectively represent the linear transformation matrix and positional embedding matrix. First, divide the input data into multiple patches, and each patch is first transformed with a linear transformation $E$ to obtain an embedding vector, which is the token. After obtaining all the tokens, add position information, i.e. ${E}^{pos}$, to these tokens, and these token sequences are the input information of the encoder.

#### 2) Transformer Encoder

Next, we input the patch with positional information into the encoder. We adopt $N$ identical encoders to extract relevant information from the sEMG signal, each consisting of a multi-head self-attention block (MSA) and a multi-layer perceptron (MLP) [28]. For ease of description, we denote the input of each layer of the encoder as ${Z}_{l}$ ($l=\text{1,2}\dots N$).

##### Multi-Head Self-Attention

The multi-head attention module is composed of multiple self-attention layers. The function of the self-attention layer is to capture the correlation between different vectors in the sequence of sEMG features, and aggregate global contextual information to update each component of the sequence.

Therefore, for a series of input segments ${Z}_{l}$, we first transform the input vectors into three different vectors: query vector $q$, key vector $k$, and value vector $v$, with dimensions of ${d}_{q}$=${d}_{k}$=${d}_{v}$=$D$. Vectors from different inputs are packed into three different matrices, namely $Q, K$, and $V$. The formula for computing the attention function between different input vectors is as follows:

8 $$\begin{array}{*{20}c} {Attention\left( {Q,K,V} \right) = softmax\left( {\frac{{Q \cdot K^{T} }}{{\sqrt {d_{k} } }}} \right) \cdot V} \\ \end{array}$$

Multi-head attention allows the model to use multiple attention heads to focus on different parts of the input sEMG patch in different ways. Assuming there are h heads, each head has a dimension of ${d}_{h}= D/h$. First, we calculate the output result ${head}_{i}$ for each attention head, then concatenate the output of multiple attention heads according to their respective dimensions and project the result into a matrix, which can be achieved through the following process:

9 $$\begin{array}{*{20}c} {head_{i} = Attention\left( {Q_{i} ,K_{i} ,V_{i} } \right)} \\ \end{array}$$

10 $$Multihead = Concat\left( {head_{1} \ldots head_{h} } \right)W$$

where ${Q}_{i}$, ${K}_{i}$, and ${V}_{i}$ respectively represent $Q, K, V$ in the calculation process of the i-th attention head, and $W \in {\mathbb{R}}^{D \times D}$ is a linear projection matrix.

To slow down the degradation of the network and accelerate the training speed, this module adds skip connections and layer normalization operations, the formula is as follows:

11 $$\begin{array}{*{20}c} {Z_{l} = Multihead\left( {LN\left( {Z_{l - 1} } \right)} \right) + Z_{l - 1} } \\ \end{array}$$

##### Multi-layer Perceptron (MLP)

The MLP consists of two linear transformation layers, a dropout layer, and a non-linear activation function called a Gaussian Error Linear Unit (GELU). The linear transformation layer of MLP maps the input data $Z$ to a higher dimensional space, helping the network integrate information. The dropout layer is used to avoid overfitting. Then filter through the nonlinear activation function, and change the data back to the original dimension after filtering. Inspired by [27], we finally chose GELU instead of the commonly used ReLU due to its higher accuracy on numerous datasets. Therefore, MLP can be described as:

12 $$\begin{array}{*{20}c} {MLP\left( {Z_{l} } \right) = W_{2} \delta \left( {W_{1} Z_{l} } \right)} \\ \end{array}$$

where ${W}_{1}$ and ${W}_{2}$ represent the parameter matrices of the two linear layers, respectively, and $\delta$ represents the activation function GELU.

As with the multi-head attention module, a residual block and a normalization layer are also added here:

13 $$\begin{array}{*{20}c} {Z^{\prime}_{l} = MLP\left( {LN\left( {Z_{l} } \right)} \right) + Z_{l} } \\ \end{array}$$

##### Prediction Layer

To avoid feature redundancy, average pooling was first used to further compress the features, and then a linear layer was used to make predictions, resulting in the predicted joint angles $y$.

14 $$\begin{array}{*{20}c} {y = Linear\left( {AvgPooling\left( {Z^{\prime}_{l} } \right)} \right)} \\ \end{array}$$

### D. Smooth Layer

Based on the preliminary results of experiments, the network predictions have certain fluctuations, which may cause the joint angle sequence to not match the smoothness of biological movements. Therefore, a smoothing module is introduced to smooth the predicted joint angle signals. The module can use a small amount of historical joint angles to handle some predicted values with larger errors, making them more consistent with actual human movements and improving the robustness of the model. The specific implementation method is described as follows:

15 $$y_{i}^{\prime } \, = \,\sum\limits_{{i = 1}}^{L} {\frac{{y_{i} }}{L}}$$

16 $$\begin{array}{*{20}c} {s_{0} = y^{\prime}_{1} + y^{\prime}_{2} + y^{\prime}_{3} } \\ \end{array}$$

17 $$\begin{array}{*{20}c} {s_{t} = \alpha y^{\prime}_{t} + \left( {1 - \alpha } \right)s_{t - 1} } \\ \end{array}$$

where $y$ is the output of MAPEN, $L$ represents the window length, and $\alpha$ represents the smoothing coefficient. According to Fig. 1. We set $L$ and $\alpha$ to 4 and 0.3, respectively.

## Experiments and results

### A. Dataset

The dataset we selected comes from the Ninapro database [2], which is a publicly available sEMG data set widely used for hand motion prediction. It contains several different sub-datasets (DB1-DB10), to ensure the diversity of subjects and movements, we choose the second database DB2 [29, 30], which contains 40 kinds of hand movements made by 40 subjects. Repeat each action 6 times, hold for 5 s, and then rest for 3 s. The gender, age, height, weight, and handedness range of the 40 subjects are as follows: 29 males/11 females, 23–45, 150–192 cm, 44–105 kg, 5 left /35 right.

During data acquisition, sEMG was recorded using a Delsys Trigno wireless system consisting of 12 wireless sEMG electrodes, and hand movements were measured using a 22-sensor Cyber Glove II. The sampling rates of Cyberglove II and Delsys are 20 and 2000 Hz respectively. To keep the two signals in sync, the first kinematics signal was resampled to 2000 Hz. We selected 40 common hand gestures as the research objects, including 8 gestures with equal length and tension, 23 grasping and functional movements, and 9 basic wrist movements (excluding hand strength testing movements). When selecting the predicted joints, we chose the proximal interphalangeal joint (PIP), metacarpophalangeal joint (MCP), and wrist joint as the estimated joints. The distal interphalangeal joint (DIP) can be easily calculated from PIP and MCP. Therefore, these 12 joints are sufficient to describe hand movements.

### B. Processing

After extracting features in the Features section, we concatenated the three different features in the channel dimension. We performed linear normalization to avoid any feature from having a substantial impact on the forecast results and induce convergence of the model. We segmented hand kinematic signals into sequences of 100 ms windows with a 0.5 ms step, similar to the processing of sEMG. The joint angle feature matrix was constructed using the last angle value within each window.

To complete the process of model training and testing, we use four out of the six repetitions performed by each subject as their training dataset, while the remaining two repetitions are allotted for testing purposes.

### C. Evaluation of Parameters

To evaluate the performance of our method and compare it with other methods, the following evaluation criteria have been introduced.

Pearson correlation coefficient (CC) is a statistical measure utilized to evaluate the strength and direction of the linear relationship between two variables [31]. In the current study, we apply CC to evaluate both the precision and consistency of the predictive model, as well as the strength of the correlation between predicted and observed results.

NRMSE is the normalized version of the root mean square error (RMSE), which is typically used to assess the degree of deviation between predicted and actual values. Unlike RMSE, the value of NRMSE is not affected by the absolute size of actual values, so it can be compared across different datasets and problems, helping us to evaluate the accuracy and reliability of predictive models.

The coefficient of determination, R^2^, is a metric utilized in measuring the accuracy of predicted data. This metric can determine the degree to which the predictive model explains the actual data, with a range between 0 and 1. A high value of R^2^ shows that the predictive model can effectively explain actual data, and there is a minimal difference between the predicted and live results [17].

$smooth$ is considered a quantitative measure for evaluating the smoothness of a curve. Here, we use it to assess the smoothness of the predicted angle signal curve, where a larger smoothing value indicates a higher estimated level of smoothness [32].

18 $$\begin{array}{*{20}c} {smooth = - \frac{1}{2n}\sum\nolimits_{i = 1}^{n - 1} {\left| {\frac{{\hat{x}\left( i \right) - \hat{x}\left( {i + 1} \right)}}{{T_{s} }}} \right|} } \\ \end{array}$$

where $\widehat{x}\left(i\right)$ are the predicted joint angles, ${T}_{s}$ is the sampling time interval, and $n$ is the total number of angle sequences.

### D. Experimental Results

The models were constructed based on the PyTorch framework [33] and were trained and evaluated on a GPU (NVIDIA GEFORCE RTX 3060) to ensure fairness during the deep learning process. Identical parameter settings such as learning rate, optimizer, and loss function were incorporated to guarantee identical training conditions. The models underwent 400 epochs of training, achieving convergence within that process. The initial learning rate was set at 0.001, which was decayed by 50% in the subsequent 200 epochs. The batch size was set at 256 for enhanced training efficiency. As for the regression task, we adopted the widely used mean squared error (MSE) as the loss function. It should be noted that the training data was shuffled to prevent overfitting.

In this paper, we conducted a comprehensive performance evaluation of various models by computing metrics including CC, RMSE, R^2^, and smoothness for each participant. Furthermore, to assess the feasibility of different methods in practical applications, we utilized RASPBERRY PI 4B as a hardware platform to measure their inference latency. The RASPBERRY PI 4B hardware has a Broadcom BCM2711 microprocessor, quad-core Cortex-A72 (ARM v8) 64-bit SoC running at 1.5 GHz, and 4 GB LPDDR4-2400 SDRAM.

To assess the performance differences between various models, we initially used the Friedman test for preliminary comparison, followed by the Wilcoxon signed-rank test for post-hoc comparisons. We adjusted the results using Bonferroni correction. The performance metrics of CC, NRMSE, and R^2^ were considered statistically significant when p < 0.05 as dependent variables.

Firstly, we conducted performance tests on the proposed sMAPEN model and compared it with simpler structures to establish the superiority of this method. We examined four structures, including MAPEN, SAPEN, MAEN, and PEN. Table 1 shows the components of five models. MAPEN removes the smoothing layer from sMAPEN. SAPEN replaces MAPEN's multi-scale attention module with a single-scale attention module that utilizes a convolutional kernel size of 7. MAEN removes the patching operation from MAPEN. PEN drops the multi-scale attention module from MAPEN.

**Table 1:** Composition of five models

Model	Attention fusion	Patch layer	Transformer encoder	Smooth layer
sMAPEN	MAF	Y	Y	Y
MAPEN	MAF	Y	Y	N
sAPEN	AF	Y	Y	Y
MAEN	MAF	N	Y	N
PEN	–	Y	Y	N

1. Y and N respectively indicate the presence and absence of this part in the model

2. MAF is the multi-scaled attention module that utilizes 3 convolutional kernel size of 3, 5 and 7 respectively.

3. AF is the single-scaled attention module that utilizes only one convolutional kernel size of 7.

4. ’s’ indicates that a smoothing layer is added to the model.

**Fig. 2:**
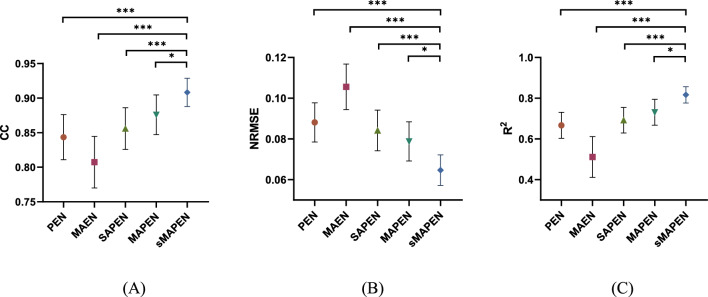
Comparison results of sMAPEN and its sub-models. **A**–**C** denote the results of CC, NRMSE, and R^2^, respectively. MAPEN removes the smoothing layer from sMAPEN. SAPEN replaces MAPEN’s multiscale attention module with a single-scale attention module that utilizes a convolutional kernel size of 7. MAEN eliminates the patching operation from MAPEN. PEN eliminates the multiscale attention module from MAPEN

The results of the comparative analysis of the predictive performance (CC, NRMSE, R^2^) of five models are presented in Fig. 2. The figure also displays the statistical analysis results. The results demonstrate that the proposed models' modules considerably enhance the predictive accuracy. To demonstrate the effectiveness of Patching, we compared MAPEN and MAEN and observed a considerable enhancement in predictive performance after incorporating Patching. Specifically, we observed a 6.9% improvement in the CC, a 0.0520 decrease in NRMSE, and a 0.2195 improvement in R^2^. In addition, we investigated the impact of the convolution module by comparing PEN, SAPEN, and MAPEN. Our results showed that compared to PEN without a convolution module, SAPEN with a single-scale convolution module exhibited a slight performance improvement. However, upgrading the single-scale module to a multi-scale convolution module led to a further improvement in performance. Specifically, we observed a 3.3% improvement in the CC, a 0.0093 decrease in NRMSE, and a 0.0638 improvement in R^2^ for MAPEN as compared to PEN, indicating that the multi-scale convolution module is capable of extracting more diverse sEMG features, thereby enhancing the overall predictive performance of the model. Lastly, we also evaluated the effectiveness of the smoothing module by comparing the performance of sMAPEN and MAPEN. Our results showed that after the addition of the smoothing module, the CC was improved by 3.2%, the NRMSE decreased by 0.0141, and the R^2^ was improved by 0.0856. We conducted a significant analysis of the performance of various models. Firstly, we conducted the Friedman test, which revealed a statistically significant difference in the performance of the methods, with a p-value less than 0.001. We then applied the Wilcoxon signed-rank test for a pairwise comparison of the methods. Apart from (sMAPEN, MAPEN), all other p-values were less than 0.001. The findings suggest that our proposed model can significantly enhance the predictive accuracy of sEMG signals and achieve a high level of performance through the refinement and optimization of the modules.

The comprehensive performance of several deep learning models in finger joint estimation is shown in Table 2, where the evaluation indicators are the average values of all joint angles and all subjects. As shown, sMAPEN achieves the best performance, with average CC, NRMSE, and R^2^ values of 0.9082 ± 0.0216, 0.0646 ± 0.0074, and 0.8163 ± 0.0398, respectively, which are significantly better than those of sBERT (0.8520 ± 0.0285, 0.0926 ± 0.0083, 0.6276 ± 0.0655), MAFN (0.8493 ± 0.0289, 0.0964 ± 0.0108, 0.6008 ± 0.0820), LSTM [32]. (0.7742 ± 0.0392, 0.0991 ± 0.0106, 0.5788 ± 0.0692), CNN-ATTENTION [34] (0.8202 ± 0.0363, 0.1030 ± 0.0101, 0.5391 ± 0.0992), and TCN (0.7421 ± 0.0414, 0.1009 ± 0.0095, 0.5529 ± 0.0639). In addition, sMAPEN's performance variance is also smaller, indicating that its performance is more stable and consistent across different subjects. Statistical analysis shows that the proposed sMAPEN model is superior to the other four deep learning models in terms of CC, NRMSE, and R2 (p-value < 0.001).

**Table 2:** Average accuracy of various models on 12 joints and 40 movements over 40 subjects

Model	CC	NRMSE	R^2^	Smooth	P-value
TCN	0.7421 ± 0.0414	0.1009 ± 0.0095	0.5529 ± 0.0639	− 2.1308 ± 0.3109	< 0.001
LSTM	0.7742 ± 0.0392	0.0991 ± 0.0106	0.5788 ± 0.0692	− 2.4003 ± 0.3277	< 0.001
CNN-attention	0.8202 ± 0.0363	0.1030 ± 0.0101	0.5391 ± 0.0992	− 2.6520 ± 0.4144	< 0.001
MAFN	0.8493 ± 0.0289	0.0964 ± 0.0108	0.6008 ± 0.0820	− 1.0691 ± 0.1577	< 0.001
sBERT	0.8520 ± 0.0285	0.0926 ± 0.0083	0.6276 ± 0.0655	− 0.9764 ± 0.1326	< 0.001
sMAPEN	0.9082 ± 0.0216	0.0646 ± 0.0074	0.8163 ± 0.0398	− 0.4545 ± 0.0712	–

1. The CNN-Attention is a convolutional model with an attention mechanism proposed in [33]

2. MAFN is the multi-attention feature fusion network proposed in [24]

3. ’s’ indicates that a smoothing layer is added to the model

4. P-VALUE refers to the significance test results of sMAPEN and this method

**Fig. 3:**
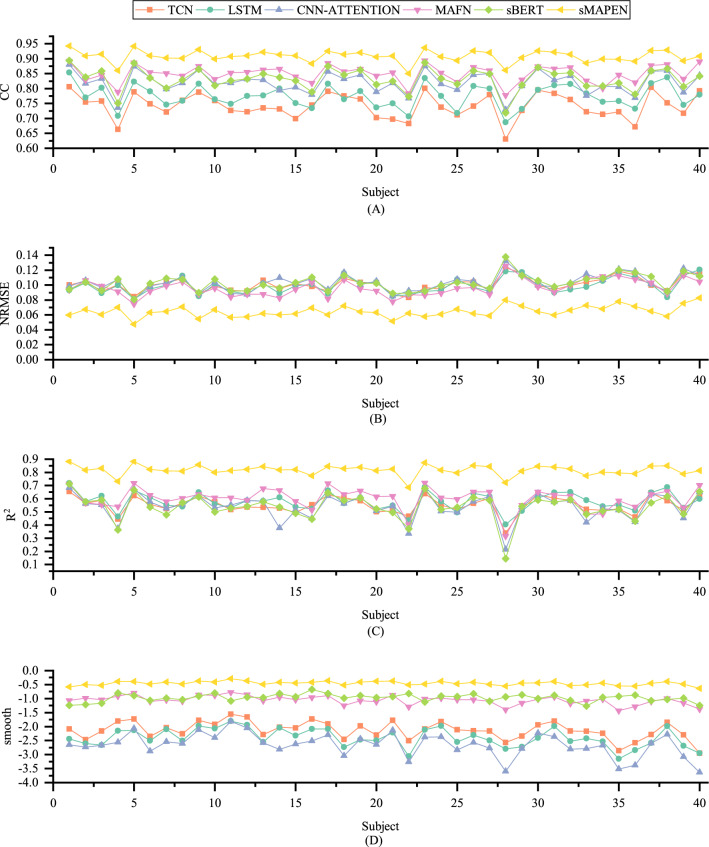
Average performance of 40 movements on 40 subjects. **A**–**D** denote the average results of CC, NRMSE, R^2^, and smooth, respectively

**Fig. 4:**
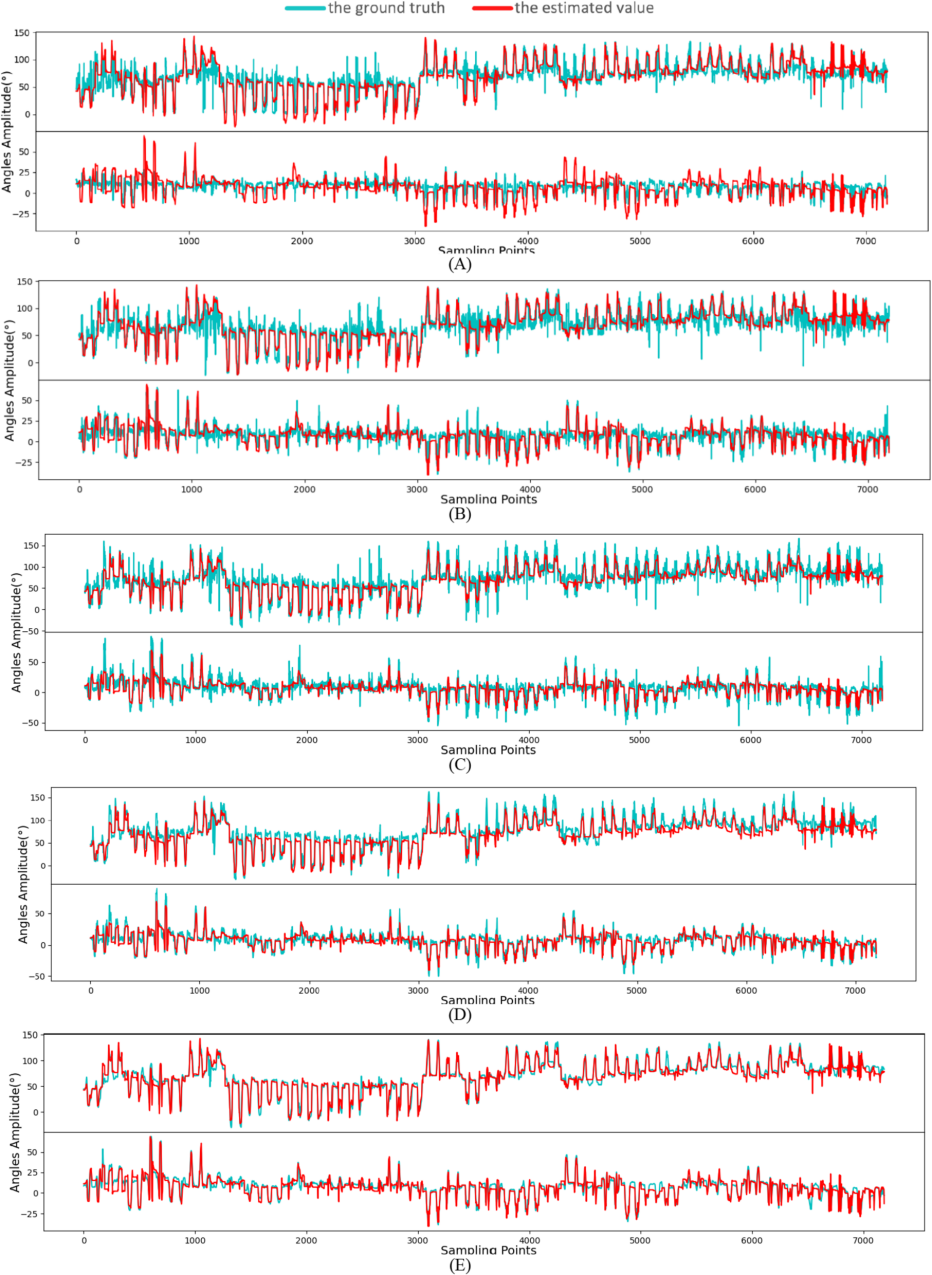
Curve chart of joint angles predicted based on sMAPEN and other methods and the actual joint angles. The red curve represents the ground truth, and the blue curve represents the estimated value. The horizontal axis represents the input features, one sampling point denotes a concatenation of features and the vertical coordinates represent the magnitude of the joint angles. Only two joints are shown in the figure to better display the estimated details, which can represent the estimation quality of all joints. **A** The actual curves of joint angles and the curves of joint angles predicted by TCN. **B** The actual curves of joint angles and the curves of joint angles predicted by LSTM. **C** The actual curves of joint angles and the curves of joint angles predicted by CNN-Attention. **D** The actual curves of joint angles and the curves of joint angles predicted by sBERT. **E** The actual curves of joint angles and the curves of joint angles predicted by sMAPEN

Then we compared the estimation accuracy of different subjects separately, as shown in Fig. 3. In Fig. 3A–C, our proposed sMAPEN provided high CC (> 0.8336), low NRMSE (< 12.6235), and high R2 (> 0.6885) for each subject, with sBERT performing second best, while the performances of CNN-Attention, LSTM, and TCN were significantly worse, especially in the predictions of the 4th and 28th subjects, where the prediction accuracy of the other models decreased significantly, but sMAPEN still maintained high accuracy, which proves its stronger generalization ability. In Fig. 3D, we have calculated the prediction smoothness of several models and found that sMAPEN performs the best (− 0.4545 ± 0.0712), slightly better than sBERT (− 0.9764 ± 0.13269) and MAFN (− 1.0691 ± 0.1577), and significantly better than CNN-ATTENTION (− 2.6520 ± 0.4144), LSTM (− 2.4003 ± 0.3277), and TCN (− 2.1308 ± 0.3109). Figure 4 provides a visualization of the prediction performance of each model, demonstrating the predictive performance of the five models on Subject 1. Compared to the other models, sMAPEN predicts joint angle curves that are more stable, accurate, and in line with the smoothness of human motion. The second place is sBERT, with basic accuracy in predicting the trend of the angle signal and relatively stable fluctuations. Although CNN-Attention, LSTM, and TCN can also predict the corresponding angle signal trend, the predicted signal fluctuations are relatively large, resulting in distortions in some of the movements.

Table 3 presents the inference time of the methods on portable devices. Inference time refers to the duration required for a model to estimate hand movements from surface electromyography signals. A longer inference time could adversely impact user experience. The results demonstrate that TCN exhibits the smallest inference latency (less than 50 ms), slightly outperforming our proposed sMAPEN (less than 100 ms), followed by CNN-Attention and MAFN. These methods can all meet the deployment performance requirements (< 200 ms) for continuous motion estimation. However, the inference time for both sBERT and LSTM exceeds 200 ms, making it challenging to meet deployment requirements in practical applications.

**Table 3:** Inference time (IT) of different models on the raspberry PI 4B

Model	Inference time (ms)
TCN	44.12
LSTM	419.96
CNN-attention	132.21
MAFN	165.87
sBERT	320.46
sMAPEN	97.93

1. The result is the average reasoning time of 40 subjects on the device

## Discussion

This paper proposes a method, sMAPEN, for estimating continuous hand movements on a large scale. We evaluated sMAPEN on 40 participants and compared its performance with four advanced hand movement estimation models. The performance of each model was quantified using various indicators, such as CC, RMSE, and R^2^, and statistically analyzed.

Based on the results provided in the previous section, our proposed method, Multi-scale Attention Patching Encoder Network (sMAPEN), demonstrated higher accuracy and stability across participants and joints than the other four models, as confirmed by statistical tests. Our analysis suggests that this may be due to the model’s memory requirements shifting from requiring less memory to needing more memory when the number of movements increased to 40. However, the Temporal Convolutional Network (TCN) method had a relatively small receptive field and could not handle the increased memory requirements, resulting in decreased performance compared to situations with fewer movements.

While LSTM has mitigated the issue of long-term dependency, it still encounters difficulties when processing longer sequences. The growth of sequence length caused by the expansion of movement combinations might degrade the estimation performance of LSTM. Both LSTM and TCN depend on preceding signal information to capture the entire sequence's global information. Yet, previous noise might impact subsequent signal samples and thereby alter the predictions made.

Compared to TCN and LSTM, CNN-Attention combines convolution with an attention mechanism to significantly improve accuracy. The same MAFN and sBERT use the multi-head attention mechanism to capture both local and global relationships in the sEMG signal in a single step, which reduces the effect of noise in the previous signal on the subsequent signal, and the accuracy of prediction is greatly improved. Our sMAPEN model has shown further improvement compared to MAFN and sBERT, possibly because it can capture local features of sEMG signals at different granularities in both time and space dimensions through the MAF module. It aggregates time steps into sEMG sub-sequence patches through a patching operation, integrating information within a segment. This further maintains the locality of the sEMG sequence. Finally, it utilizes a multi-head attention mechanism to capture comprehensive semantic information that is not available at the point level. It is worth noting that we captured the local and global relationships of sEMG signals in a single step, without being affected by previous noise. Additionally, the smoothing module plays a crucial role in achieving exceptional performance. It corrects angle prediction errors by utilizing previously predicted joint angles, resulting in smoother angle signals that demonstrate greater consistency with actual human motion.

After comparing the performance of the models, we evaluated their inference latency on mobile devices. The results showed that TCN had the lowest inference cost due to its convolutional structure and the least number of parameters. Following that were sMAPEN, CNN-Attention, and MAFN, which both supported parallel computing and had low latency. In contrast, due to the larger number of parameters, the inference latency of sBERT increased significantly. Additionally, the unique recurrent structure of LSTM hindered parallel computing, resulting in relatively high inference costs, which did not meet the deployment requirement.

In practice, the changes in skin condition and electrode placement may alter the data distribution, which may reduce the accuracy of the model. To investigate the robustness of the model, we conducted artificial simulations to test the impact of noise on the prediction results. During the simulation experiment, we included Gaussian white noise in the test dataset, with a signal-to-noise ratio of 20 dB. The outcomes depict that, despite adding noise, the sMAPEN model managed to maintain an average accuracy of 0.88, exhibiting a decrease of less than 3% in contrast to other models, which demonstrated a reduction of more than 5%. The results suggest that sMAPEN presents enhanced robustness when compared to other models.

Our model has some limitations. Firstly, we only selected healthy participants as experimental subjects, which could introduce potential bias and may not guarantee the predictive performance of the model for people with disabilities, leading to a lack of validation of the model’s generalizability. Secondly, the model’s robustness was only validated by artificially adding simulated noise, and this method of validation is not comprehensive. In our future work, we plan to conduct further research to assess the impact of various factors, such as the different types of noise, electrode displacements, and arm position on the model. Besides, although the robustness of the model was verified through manual noise simulation in this article, this verification method is not comprehensive. In the future, we will strive to include more types of noise and more complex scenarios to evaluate the robustness of the model comprehensively. In addition, there may be user differences in practical applications, and different users may have different operating habits or physiological characteristics, which may affect the model’s performance. In future research, we will design models to adapt to the behavior of different users [37], which can be achieved through online learning or incremental learning. The model will continuously learn and improve from user usage.

## Conclusion

In this study, we propose a lightweight model called sMAPEN while accurately estimating the continuous hand movements, it can also maintain compatibility with multiple movements. A multi-scale convolutional fusion module was designed specifically to extract multi-scale temporal and spatial information from the sEMG. Furthermore, a multi-head attention mechanism was also added to extract global information from the sEMG. Conducting patch operations on the sEMG contributed significantly to enhancing the accuracy of prediction and reducing the number of model parameters. We also incorporated a smoothing module which improved the model’s performance in generating realistic predicted angle signals. To assess our proposed sMAPEN, we select several representative methods (TCN, LSTM, CNN, MAFN, and sBERT) to benchmark with the proposed sMAFN. The experimental results indicate that sMAFN outperformed the alternatives by providing higher accuracy and stability while maintaining a fast speed. We extended the predicted movements to 40 types, which covered most of the static hand gestures and functional movements in our daily lives. The latency of this algorithm is only 97.93 ms, which meets the practical application requirements of wearable devices. These results demonstrate sMAPEN has significant potential in the field of HMI.

## Data Availability

All data analyzed during the current study are publicly available in the NinaPro repository, http://ninapro.hevs.ch/.
